# Statistical Report of the Wills Hospital for Diseases of the Eye and Limbs

**Published:** 1839-04-13

**Authors:** 


*Statistical Report of the Wills Hospital for Diseases of the Eye and Limbs.*

| | No. of Cases. | No. Cured. | No. Relieved. | No. Removed. | No. Incurable. | No. in the House. |
|---|---|---|---|---|---|---|
| I. Of the Eye. | | | | | | |
| Amaurosis, | 23 | 6 | 5 | 4 | 7 | 1 |
| Cataract, | 24 | 9 | 7 | 3 | 4 | 1 |
| Conjunctivitis, | 12 | 11 | 0 | 0 | 1 | 0 |
| Cornea, Opacity of | 7 | 1 | 3 | 0 | 1 | 2 |
| Cornea, Ulcer of. | 11 | 9 | 0 | 1 | 1 | 0 |
| Corneitis, Scrof. | 2 | 2 | 0 | 0 | 0 | 0 |
| Inj. and Wounds of Eye, | 5 | 4 | 1 | 0 | 0 | 0 |
| Iritis, | 12 | 8 | 3 | 0 | 1 | 0 |
| Lagopthalmus, | 1 | 0 | 1 | 0 | 0 | 0 |
| Lippitudo, | 2 | 1 | 1 | 0 | 0 | 0 |
| Nebula, | 5 | 3 | 2 | 0 | 0 | 0 |
| Obstr. Lachr. Duct. | 2 | 1 | 1 | 0 | 0 | 0 |
| Ophthalmia, | 55 | 46 | 9 | 0 | 0 | 0 |
| " Catarrhal, | 1 | 1 | 0 | 0 | 0 | 0 |
| " Chronic, | 16 | 13 | 2 | 0 | 0 | 1 |
| " Granular, | 10 | 8 | 1 | 0 | 0 | 1 |
| " Leucorrheal, | 1 | 0 | 1 | 0 | 0 | 0 |
| " Purulent, | 5 | 2 | 2 | 1 | 0 | 0 |
| " Pustular, | 1 | 1 | 0 | 0 | 0 | 0 |
| " Rheumatic, | 6 | 5 | 0 | 0 | 0 | 1 |
| " Scrofulous, | 27 | 22 | 3 | 2 | 0 | 0 |
| " Tarsal, | 12 | 8 | 3 | 0 | 0 | 1 |
| | 240 | 161 | 45 | 11 | 15 | 8 |
| II. Of the Limbs. | | | | | | |
| Abscesses, | 4 | 0 | 2 | 2 | 0 | 0 |
| Chilblains, | 1 | 1 | 0 | 0 | 0 | 0 |
| Coxalgia, | 1 | 0 | 0 | 1 | 0 | 0 |
| Disease of Joints, | 8 | 3 | 3 | 3 | 0 | 0 |
| Fractures, | 5 | 5 | 0 | 0 | 0 | 0 |
| Gout, | 1 | 1 | 0 | 0 | 0 | 0 |
| Paralysis, | 2 | 1 | 0 | 0 | 1 | 0 |
| Rheumatism, | 13 | 10 | 1 | 1 | 1 | 0 |
| Ulcers, | 5 | 3 | 0 | 1 | 1 | 0 |
| Wounds, | 3 | 2 | 1 | 0 | 0 | 0 |
| | 44 | 26 | 7 | 8 | 3 | 0 |
| Total, | 284 | 187 | 52 | 19 | 18 | 8 |

| | No. of Cases. | No. Cured. | No. Relieved. | No. Removed.* | No. Incurable. | No. in the house. |
|---|---|---|---|---|---|---|
| 1834, | 31 | 18 | 8 | 2 | 3 | 0 |
| 1835, | 49 | 27 | 15 | 5 | 2 | 0 |
| 1836, | 51 | 39 | 7 | 1 | 4 | 0 |
| 1837, | 66 | 40 | 15 | 5 | 5 | 1 |
| 1838, | 87 | 63 | 7 | 6 | 4 | 7 |

**Removed.* Those in whom the treatment was not completed. They were either removed by friends, discharged as disorderly, eloped, or died.

